# Methyl 3′,5′-dimeth­oxy­biphenyl-4-carboxyl­ate

**DOI:** 10.1107/S1600536813005333

**Published:** 2013-02-28

**Authors:** Manu Lahtinen, Kalle Nättinen, Sami Nummelin

**Affiliations:** a University of Jyväskylä, Department of Chemistry, PO Box 35, FI-40014 JY, Finland; b VTT Technical Research Centre of Finland, Tampere, FIN-33101, Finland; c Molecular Materials, Department of Applied Physics, School of Science, Aalto University, PO Box 15100, FI-00076 Aalto, Finland

## Abstract

In the title compound, C_16_H_16_O_4_, the dihedral angle between the benzene rings is 28.9 (2)°. In the crystal, mol­ecules are packed in layers parallel to the *b* axis in which they are connected *via* weak inter­molecular C—H⋯O contacts. Face-to-face π–π inter­actions also exist between the benzene rings of adjacent mol­ecules, with centroid–centroid and plane-to-plane shift distances of 3.8597 (14) and 1.843 (2) Å, respectively.

## Related literature

For related structures, see: Lahtinen *et al.* (2013 ▶); Van Eerdenbrugh *et al.* (2010 ▶). For the nature of hydrogen bonding, see: Steiner (2002 ▶). For synthesis details and related supra­molecular structures based on biphenyls, see: Percec *et al.* (2006 ▶, 2007 ▶). For related synthetic biphenyl structures, see: Rosen *et al.* (2008 ▶); Percec *et al.* (2004 ▶); Wolfe *et al.* (1999 ▶). For polyester functionalized dendrons and dendrimers, see: Nummelin *et al.* (2000 ▶). For a general review on self-assembling dendrons and dendrimers, see: Rosen *et al.* (2009 ▶). 
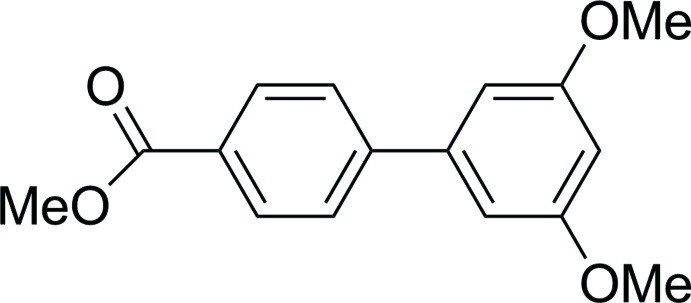


## Experimental

### 

#### Crystal data

C_16_H_16_O_4_
*M* *_r_* = 272.29
Triclinic, 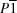

*a* = 6.0990 (9) Å
*b* = 7.1622 (16) Å
*c* = 16.2408 (18) Å
α = 96.589 (14)°
β = 91.472 (11)°
γ = 112.493 (18)°
*V* = 649.27 (19) Å^3^
*Z* = 2
Cu *K*α radiation
μ = 0.82 mm^−1^
*T* = 123 K
0.29 × 0.19 × 0.04 mm

#### Data collection

Agilent SuperNova (Dual, Cu at zero, Atlas) diffractometer
Absorption correction: analytical *CrysAlis PRO*; Agilent, 2010 ▶) *T*_min_ = 0.924, *T*_max_ = 0.983
3989 measured reflections
2395 independent reflections
2025 reflections with *I* > 2σ(*I*)
*R*_int_ = 0.025

#### Refinement

*R*[*F* ^2^ > 2σ(*F* ^2^)] = 0.040
*wR*(*F* ^2^) = 0.111
*S* = 1.05
2395 reflections
184 parameters
H-atom parameters constrained
Δρ_max_ = 0.19 e Å^−3^
Δρ_min_ = −0.25 e Å^−3^

### 

Data collection: *CrysAlis PRO* (Agilent, 2010 ▶); cell refinement: *CrysAlis PRO*; data reduction: *CrysAlis PRO*; program(s) used to solve structure: *SUPERFLIP* (Palatinus & Chapuis, 2007 ▶); program(s) used to refine structure: *SHELXL97* (Sheldrick, 2008 ▶); molecular graphics: *OLEX2* (Dolomanov *et al.*, 2009 ▶) and *Mercury* (Macrae *et al.*, 2006 ▶); software used to prepare material for publication: *OLEX2*.

## Supplementary Material

Crystal structure: contains datablock(s) I, global. DOI: 10.1107/S1600536813005333/fj2616sup1.cif

Structure factors: contains datablock(s) I. DOI: 10.1107/S1600536813005333/fj2616Isup2.hkl

Supplementary material file. DOI: 10.1107/S1600536813005333/fj2616Isup3.cml

Additional supplementary materials:  crystallographic information; 3D view; checkCIF report

## Figures and Tables

**Table 1:** Hydrogen-bond geometry (Å, °)

*D*—H⋯*A*	*D*—H	H⋯*A*	*D*⋯*A*	*D*—H⋯*A*
C6—H6⋯O2^i^	0.95	2.89	3.7702 (19)	154
C7—H7⋯O4^ii^	0.95	2.85	3.4607 (19)	123
C12—H12⋯O4^ii^	0.95	2.71	3.644 (2)	170
C16—H16⋯O14^iii^	0.95	2.65	3.514 (2)	151
C1—H1*A*⋯O4^iv^	0.98	2.73	3.665 (2)	159
C19—H19*A*⋯O18^v^	0.98	2.65	3.538 (2)	151

Symmetry codes: (i) 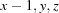; (ii) 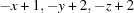; (iii) 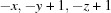; (iv) 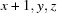; (v) 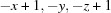.

**Fig. 1.**
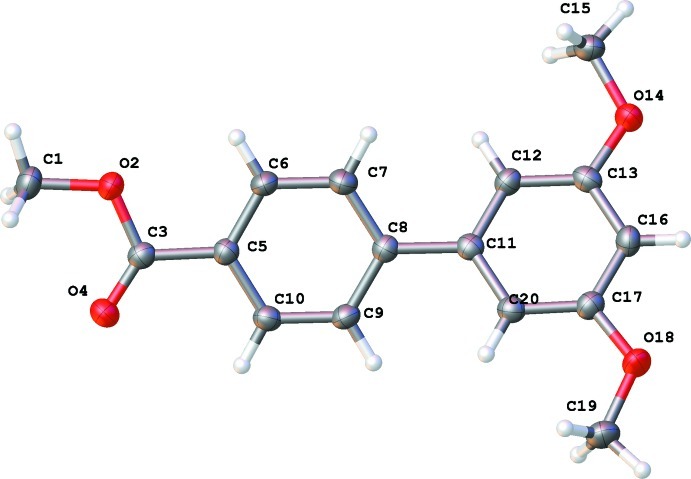
The molecular structure and labelling of the title compound. The displacement ellipsoids are drawn with 50% probability.

**Fig. 2.**
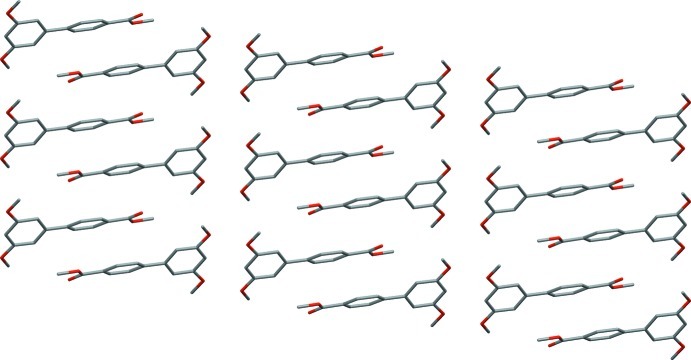
Packing scheme along a-axis.

**Fig. 3.**
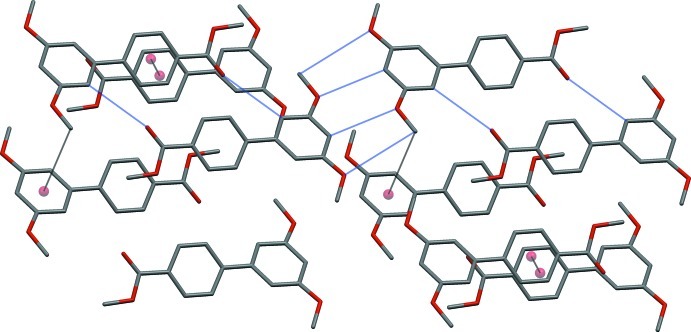
Weak CH···O (blue lines), CH···π and π···π interactions occurring in the structure.

## supplementary crystallographic information

### Comment

Biphenyls represent a new class of arylmethyl ethers and esters that serve as building blocks for the construction of amphiphilic dendrons. These dendrons, synthesized in a multi-step reaction sequence, self-assemble into hollow and non-hollow supramolecular dendrimers that further self-organize into periodic assemblies (Percec *et al.* 2006, 2007). The key synthetic step is the C—C bond formation between the aromatic rings. This is accomplished with metal catalyzed cross-coupling reaction (Percec *et al.* 2004, 2006; Rosen *et al.* 2008, Wolfe *et al.* 1999). As a contribution to a structural study of biphenyl ester derivatives we report here the title compound methyl-(3',5'-dimethoxy-biphenyl)-4-carboxylate (I).

Compound (I) crystallizes in triclinic space group *P*-1 (No. 2) without any solvent molecules, and having a single molecule in an asymmetric unit (Fig. 1). The intramolecular dihedral angle between the phenyl rings is 28.9 (2)°, similar to that of reported for analogous methyl 3',4',5'-trimethoxybiphenyl-4- carboxylate (Lahtinen *et al.* 2013). The molecules are packed in antiparallel arrangement (Fig. 2). Weak intermolecular π-π interactions (face-to-face) exist between adjacent aromatic rings having centroid-centroid and plane to plane shift distances of 3.8597 (14) and 1.843 (2) Å, respectively. Whereas weak CH-π interaction occurs between a methoxy group and nearby phenyl ring with a d(D···centroid) of 3.8303 (19) Å. Additionally, weak intermolecular CH···O hydrogen bonds occur between aromatic H atoms and neighboring methoxy O atoms with d(D···A) varying between 3.4607 (19), and 3.7702 (19) Å. Moreover, weak hydrogen bond exist between the carbonyl oxygen and aromatic ring hydrogen (C12—H12···O4) with d(D···A) of 3.644 (2) Å. The methyl ester and the two methoxy groups show characteristic geometries, typical bond distances and angles for these groups (see Tables) and are in planar orientation towards their host rings.

### Experimental

3',5'-dimethoxy-biphenyl-4-carboxylic acid (15.50 g, 60.00 mmol) was dissolved in methanol (300 ml) and sulfuric acid (3 ml). The solution was stirred under reflux for 16 h and then allowed to cool down to room temperature. Water (600 ml) was added, the resulting precipitate was collected by suction filtration. The solid was washed with water, dried in *vacuo* affording the title compound as a white crystalline solid (15.69 g, 96%). Single-crystals were obtained from a slow evaporation of ethanol.

### Refinement

Hydrogen atoms were calculated to their positions as riding atoms (C host) using isotropic displacement parameters that were fixed to be 1.2 or 1.5 times larger than those of the attached non-hydrogen atom.

### Crystal data

**Table:**

C_16_H_16_O_4_	*Z* = 2
*M**_r_* = 272.29	*F*(000) = 288
Triclinic, *P*1	*D*_x_ = 1.393 Mg m^−^^3^
*a* = 6.0990 (9) Å	Cu *K*α radiation, λ = 1.5418 Å
*b* = 7.1622 (16) Å	Cell parameters from 2092 reflections
*c* = 16.2408 (18) Å	θ = 5.5–76.0°
α = 96.589 (14)°	µ = 0.82 mm^−^^1^
β = 91.472 (11)°	*T* = 123 K
γ = 112.493 (18)°	Plate, colourless
*V* = 649.27 (19) Å^3^	0.29 × 0.19 × 0.04 mm

### Data collection

**Table:**

Agilent SuperNova (Dual, Cu at zero, Atlas) diffractometer	2395 independent reflections
Radiation source: SuperNova (Cu) X-ray Source	2025 reflections with *I* > 2σ(*I*)
Mirror monochromator	*R*_int_ = 0.025
Detector resolution: 10.3953 pixels mm^-1^	θ_max_ = 69.0°, θ_min_ = 5.5°
ω scans	*h* = −5→7
Absorption correction: analytical *CrysAlis PRO*; Agilent, 2010)	*k* = −8→8
*T*_min_ = 0.924, *T*_max_ = 0.983	*l* = −19→19
3989 measured reflections	

### Refinement

**Table:**

Refinement on *F*^2^	Primary atom site location: structure-invariant direct methods
Least-squares matrix: full	Secondary atom site location: difference Fourier map
*R*[*F*^2^ > 2σ(*F*^2^)] = 0.040	Hydrogen site location: inferred from neighbouring sites
*wR*(*F*^2^) = 0.111	H-atom parameters constrained
*S* = 1.05	*w* = 1/[σ^2^(*F*_o_^2^) + (0.0591*P*)^2^ + 0.1033*P*] where *P* = (*F*_o_^2^ + 2*F*_c_^2^)/3
2395 reflections	(Δ/σ)_max_ = 0.001
184 parameters	Δρ_max_ = 0.19 e Å^−^^3^
0 restraints	Δρ_min_ = −0.25 e Å^−^^3^

### Special details

**Table:**

Geometry. All e.s.d.'s (except the e.s.d. in the dihedral angle between two l.s. planes) are estimated using the full covariance matrix. The cell e.s.d.'s are taken into account individually in the estimation of e.s.d.'s in distances, angles and torsion angles; correlations between e.s.d.'s in cell parameters are only used when they are defined by crystal symmetry. An approximate (isotropic) treatment of cell e.s.d.'s is used for estimating e.s.d.'s involving l.s. planes.
Refinement. Refinement of *F*^2^ against ALL reflections. The weighted *R*-factor *wR* and goodness of fit *S* are based on *F*^2^, conventional *R*-factors *R* are based on *F*, with *F* set to zero for negative *F*^2^. The threshold expression of *F*^2^ > σ(*F*^2^) is used only for calculating *R*-factors(gt) *etc*. and is not relevant to the choice of reflections for refinement. *R*-factors based on *F*^2^ are statistically about twice as large as those based on *F*, and *R*- factors based on ALL data will be even larger.

### Fractional atomic coordinates and isotropic or equivalent isotropic displacement parameters (Å^2^)

**Table:**

	*x*	*y*	*z*	*U*_iso_*/*U*_eq_	
O4	0.72065 (19)	0.88715 (17)	1.15112 (6)	0.0256 (3)	
O2	1.01438 (18)	0.78669 (16)	1.11239 (6)	0.0245 (3)	
O18	0.46733 (19)	0.23876 (16)	0.56673 (6)	0.0256 (3)	
O14	0.1145 (2)	0.71333 (18)	0.59191 (7)	0.0301 (3)	
C3	0.8129 (3)	0.8161 (2)	1.09788 (9)	0.0205 (3)	
C6	0.5135 (3)	0.7715 (2)	0.98314 (9)	0.0210 (3)	
H6	0.4281	0.8202	1.0223	0.025*	
C17	0.4269 (3)	0.3845 (2)	0.61901 (9)	0.0213 (3)	
C13	0.2432 (3)	0.6276 (2)	0.63282 (9)	0.0220 (3)	
C7	0.4295 (3)	0.7185 (2)	0.89982 (9)	0.0212 (3)	
H7	0.2877	0.7335	0.8827	0.025*	
C11	0.4573 (2)	0.5869 (2)	0.75182 (9)	0.0201 (3)	
C5	0.7225 (3)	0.7530 (2)	1.00911 (9)	0.0202 (3)	
C12	0.3244 (3)	0.6839 (2)	0.71684 (9)	0.0214 (3)	
H12	0.2899	0.7865	0.7497	0.026*	
C16	0.2934 (3)	0.4787 (2)	0.58389 (9)	0.0226 (3)	
H16	0.2370	0.4417	0.5269	0.027*	
C8	0.5486 (2)	0.6439 (2)	0.84072 (9)	0.0196 (3)	
C9	0.7577 (3)	0.6253 (2)	0.86810 (9)	0.0223 (3)	
H9	0.8427	0.5753	0.8292	0.027*	
C20	0.5067 (3)	0.4362 (2)	0.70285 (9)	0.0211 (3)	
H20	0.5948	0.3689	0.7268	0.025*	
C10	0.8431 (3)	0.6785 (2)	0.95095 (9)	0.0222 (3)	
H10	0.9851	0.6639	0.9682	0.027*	
C1	1.1197 (3)	0.8460 (2)	1.19672 (9)	0.0255 (3)	
H1A	1.2785	0.8419	1.1987	0.038*	
H1B	1.0194	0.7518	1.2325	0.038*	
H1C	1.1328	0.9848	1.2163	0.038*	
C19	0.6415 (3)	0.1676 (3)	0.59536 (10)	0.0271 (3)	
H19A	0.6742	0.0828	0.5495	0.041*	
H19B	0.5810	0.0866	0.6406	0.041*	
H19C	0.7884	0.2845	0.6156	0.041*	
C15	0.0299 (3)	0.8481 (3)	0.64007 (10)	0.0279 (4)	
H15A	−0.0638	0.8943	0.6035	0.042*	
H15B	0.1656	0.9662	0.6678	0.042*	
H15C	−0.0700	0.7764	0.6820	0.042*	

### Atomic displacement parameters (Å^2^)

**Table:**

	*U*^11^	*U*^22^	*U*^33^	*U*^12^	*U*^13^	*U*^23^
O4	0.0290 (6)	0.0305 (6)	0.0189 (5)	0.0141 (5)	0.0027 (4)	−0.0002 (4)
O2	0.0254 (6)	0.0301 (6)	0.0191 (5)	0.0135 (5)	−0.0029 (4)	−0.0018 (4)
O18	0.0309 (6)	0.0269 (6)	0.0208 (5)	0.0150 (5)	−0.0011 (4)	−0.0025 (4)
O14	0.0377 (6)	0.0365 (6)	0.0232 (6)	0.0235 (5)	−0.0032 (5)	0.0010 (5)
C3	0.0208 (7)	0.0181 (7)	0.0214 (7)	0.0059 (6)	0.0011 (6)	0.0039 (6)
C6	0.0231 (7)	0.0198 (7)	0.0201 (7)	0.0084 (6)	0.0038 (6)	0.0012 (6)
C17	0.0219 (7)	0.0198 (7)	0.0201 (7)	0.0064 (6)	0.0033 (6)	0.0008 (6)
C13	0.0217 (7)	0.0231 (8)	0.0218 (7)	0.0087 (6)	0.0004 (6)	0.0051 (6)
C7	0.0213 (7)	0.0232 (8)	0.0199 (7)	0.0094 (6)	0.0013 (6)	0.0036 (6)
C11	0.0181 (7)	0.0202 (7)	0.0194 (7)	0.0044 (6)	0.0026 (6)	0.0034 (6)
C5	0.0221 (7)	0.0182 (7)	0.0187 (7)	0.0060 (6)	0.0019 (6)	0.0029 (5)
C12	0.0211 (7)	0.0221 (8)	0.0202 (7)	0.0079 (6)	0.0013 (6)	0.0015 (6)
C16	0.0246 (8)	0.0237 (8)	0.0180 (7)	0.0081 (6)	0.0006 (6)	0.0017 (6)
C8	0.0202 (7)	0.0169 (7)	0.0199 (7)	0.0050 (6)	0.0021 (6)	0.0029 (5)
C9	0.0223 (7)	0.0254 (8)	0.0192 (7)	0.0099 (6)	0.0033 (6)	0.0006 (6)
C20	0.0208 (7)	0.0211 (7)	0.0210 (7)	0.0077 (6)	0.0007 (6)	0.0031 (6)
C10	0.0195 (7)	0.0242 (8)	0.0229 (8)	0.0091 (6)	0.0011 (6)	0.0019 (6)
C1	0.0276 (8)	0.0288 (8)	0.0190 (7)	0.0107 (7)	−0.0046 (6)	0.0008 (6)
C19	0.0272 (8)	0.0302 (8)	0.0258 (8)	0.0147 (7)	0.0010 (6)	−0.0017 (6)
C15	0.0299 (8)	0.0294 (8)	0.0284 (8)	0.0164 (7)	0.0004 (6)	0.0021 (6)

### Geometric parameters (Å, º)

**Table:**

O4—C3	1.2107 (18)	C11—C20	1.397 (2)
O2—C3	1.3436 (17)	C5—C10	1.392 (2)
O2—C1	1.4427 (17)	C12—H12	0.9500
O18—C17	1.3694 (17)	C16—H16	0.9500
O18—C19	1.4308 (18)	C8—C9	1.399 (2)
O14—C13	1.3676 (18)	C9—H9	0.9500
O14—C15	1.4278 (18)	C9—C10	1.386 (2)
C3—C5	1.485 (2)	C20—H20	0.9500
C6—H6	0.9500	C10—H10	0.9500
C6—C7	1.391 (2)	C1—H1A	0.9800
C6—C5	1.391 (2)	C1—H1B	0.9800
C17—C16	1.389 (2)	C1—H1C	0.9800
C17—C20	1.393 (2)	C19—H19A	0.9800
C13—C12	1.399 (2)	C19—H19B	0.9800
C13—C16	1.389 (2)	C19—H19C	0.9800
C7—H7	0.9500	C15—H15A	0.9800
C7—C8	1.396 (2)	C15—H15B	0.9800
C11—C12	1.400 (2)	C15—H15C	0.9800
C11—C8	1.487 (2)		
			
C3—O2—C1	115.97 (11)	C7—C8—C11	121.35 (13)
C17—O18—C19	117.27 (11)	C7—C8—C9	117.57 (13)
C13—O14—C15	117.93 (12)	C9—C8—C11	121.09 (13)
O4—C3—O2	123.42 (13)	C8—C9—H9	119.4
O4—C3—C5	125.19 (13)	C10—C9—C8	121.19 (13)
O2—C3—C5	111.38 (12)	C10—C9—H9	119.4
C7—C6—H6	120.0	C17—C20—C11	120.10 (13)
C5—C6—H6	120.0	C17—C20—H20	119.9
C5—C6—C7	119.98 (13)	C11—C20—H20	119.9
O18—C17—C16	115.76 (13)	C5—C10—H10	119.7
O18—C17—C20	123.83 (13)	C9—C10—C5	120.53 (14)
C16—C17—C20	120.40 (13)	C9—C10—H10	119.7
O14—C13—C12	124.10 (13)	O2—C1—H1A	109.5
O14—C13—C16	114.75 (13)	O2—C1—H1B	109.5
C16—C13—C12	121.14 (13)	O2—C1—H1C	109.5
C6—C7—H7	119.2	H1A—C1—H1B	109.5
C6—C7—C8	121.62 (13)	H1A—C1—H1C	109.5
C8—C7—H7	119.2	H1B—C1—H1C	109.5
C12—C11—C8	120.57 (13)	O18—C19—H19A	109.5
C20—C11—C12	119.93 (14)	O18—C19—H19B	109.5
C20—C11—C8	119.50 (13)	O18—C19—H19C	109.5
C6—C5—C3	119.12 (13)	H19A—C19—H19B	109.5
C6—C5—C10	119.11 (14)	H19A—C19—H19C	109.5
C10—C5—C3	121.77 (13)	H19B—C19—H19C	109.5
C13—C12—C11	119.01 (13)	O14—C15—H15A	109.5
C13—C12—H12	120.5	O14—C15—H15B	109.5
C11—C12—H12	120.5	O14—C15—H15C	109.5
C17—C16—C13	119.41 (14)	H15A—C15—H15B	109.5
C17—C16—H16	120.3	H15A—C15—H15C	109.5
C13—C16—H16	120.3	H15B—C15—H15C	109.5
			
O4—C3—C5—C6	−1.2 (2)	C12—C11—C8—C7	28.9 (2)
O4—C3—C5—C10	177.90 (14)	C12—C11—C8—C9	−150.86 (14)
O2—C3—C5—C6	179.48 (13)	C12—C11—C20—C17	0.9 (2)
O2—C3—C5—C10	−1.4 (2)	C16—C17—C20—C11	−1.4 (2)
O18—C17—C16—C13	179.66 (13)	C16—C13—C12—C11	−0.4 (2)
O18—C17—C20—C11	−179.97 (13)	C8—C11—C12—C13	179.45 (13)
O14—C13—C12—C11	−179.23 (13)	C8—C11—C20—C17	−178.56 (13)
O14—C13—C16—C17	178.85 (13)	C8—C9—C10—C5	−0.3 (2)
C3—C5—C10—C9	−178.42 (14)	C20—C17—C16—C13	1.0 (2)
C6—C7—C8—C11	179.75 (13)	C20—C11—C12—C13	0.0 (2)
C6—C7—C8—C9	−0.4 (2)	C20—C11—C8—C7	−151.58 (14)
C6—C5—C10—C9	0.7 (2)	C20—C11—C8—C9	28.6 (2)
C7—C6—C5—C3	178.20 (13)	C1—O2—C3—O4	−0.2 (2)
C7—C6—C5—C10	−0.9 (2)	C1—O2—C3—C5	179.10 (12)
C7—C8—C9—C10	0.2 (2)	C19—O18—C17—C16	167.89 (13)
C11—C8—C9—C10	179.99 (13)	C19—O18—C17—C20	−13.5 (2)
C5—C6—C7—C8	0.8 (2)	C15—O14—C13—C12	−9.0 (2)
C12—C13—C16—C17	−0.1 (2)	C15—O14—C13—C16	172.12 (13)

### Hydrogen-bond geometry (Å, º)

**Table:**

*D*—H···*A*	*D*—H	H···*A*	*D*···*A*	*D*—H···*A*
C6—H6···O2^i^	0.95	2.89	3.7702 (19)	154
C7—H7···O4^ii^	0.95	2.85	3.4607 (19)	123
C12—H12···O4^ii^	0.95	2.71	3.644 (2)	170
C16—H16···O14^iii^	0.95	2.65	3.514 (2)	151
C1—H1*A*···O4^iv^	0.98	2.73	3.665 (2)	159
C19—H19*A*···O18^v^	0.98	2.65	3.538 (2)	151

Symmetry codes: (i) *x*−1, *y*, *z*; (ii) −*x*+1, −*y*+2, −*z*+2; (iii) −*x*, −*y*+1, −*z*+1; (iv) *x*+1, *y*, *z*; (v) −*x*+1, −*y*, −*z*+1.
